# Heat Shock Proteins (HSPs) and Cardiovascular Complications of Obesity: Searching for Potential Biomarkers

**DOI:** 10.3390/cimb45120588

**Published:** 2023-11-23

**Authors:** Yuriy S. Timofeev, Anton R. Kiselev, Olga N. Dzhioeva, Oxana M. Drapkina

**Affiliations:** National Medical Research Center for Therapy and Preventive Medicine, 101990 Moscow, Russia

**Keywords:** heat shock protein, HSP, HSP27, HSP60, HSP70, obesity, cardiovascular diseases, diabetes, insulin resistance, hypertension

## Abstract

Heat shock proteins (HSPs), a family of proteins that support cellular proteostasis and perform a protective function under various stress conditions, such as high temperature, intoxication, inflammation, or tissue hypoxia, constitute a promising group of possible biochemical markers for obesity and cardiovascular diseases. HSP27 is involved in essential cellular processes occurring in conditions of obesity and its cardiometabolic complications; it has protective properties, and its secretion may indicate a cellular response to stress. HSP40 plays a controversial role in the pathogenesis of obesity. HSP60 is involved in various pathological processes of the cardiovascular, immune, excretory, and nervous systems and is associated with obesity and concomitant diseases. The hypersecretion of HSP60 is associated with poor prognosis; hence, this protein may become a target for further research on obesity and its cardiovascular complications. According to most studies, intracellular HSP70 is an obesity-promoting factor, whereas extracellular HSP70 exhibited inconsistent dynamics across different patient groups and diagnoses. HSPs are involved in the pathogenesis of cardiovascular pathology. However, in the context of cardiovascular and metabolic pathology, these proteins require further investigation.

## 1. Introduction

Obesity is a chronic multifactorial disease that manifests in the excessive accumulation of adipose tissue accompanied by an increased risk of cardiovascular events, complications, and comorbidities [1,2].

The process of excessive adipose tissue formation is associated with the development of cardiovascular diseases and metabolic changes, the specific pathogenetic mechanisms of which are not yet fully understood [3,4,5]. Due to the complexity of the problem, it is appropriate to consider obesity as an interdisciplinary issue studied by cardiologists, endocrinologists, gastroenterologists, along with specialists in functional, instrumental, and clinical/laboratory diagnosis [1,6]. Biochemical laboratory diagnosis is among the top noninvasive methods of examining and studying biomarkers in circulating blood; it allows for identifying biochemical changes in the human body [7].

Accordingly, the search for potential biomarkers remains an urgent task in laboratory and clinical science. One of the promising groups of biochemical factors includes heat shock proteins (HSPs), a family of proteins that plays an important role at the molecular, cellular, tissue, and organ system levels [8]. There is now much evidence regarding the role of HSPs in cardiovascular disease, but less is known about their association with obesity.

Hence, the objective of this review is to describe, systematize, and analyze the contemporary published data from experimental and clinical studies on the relationships between HSPs, obesity, and cardiovascular diseases.

## 2. HSP Family: Biochemistry and Clinical Significance

HSPs constitute a ubiquitous and conserved protein family in both prokaryotic and eukaryotic organisms. They maintain cellular proteostasis and, under physiological conditions, have a protective function against cellular stress. A key feature of HSPs is their increased expression under various stress conditions, such as high temperature, intoxication, inflammatory response, or tissue hypoxia [9].

HSPs were discovered in the middle of the 20th century, when the activation of the processes of specific protein group synthesis in *Drosophila* larvae in response to a temperature increase was discovered. Such association with the response to elevated temperature gave rise to the name of this group of molecules: heat shock proteins [10]. Further studies showed that the conditions for HSP synthesis were very general and, in addition to increased temperature, they involved other stressors, such as solvents, heavy metals, oxidants, hormones, and growth factors. Thus, in a broader sense, HSPs can be considered as biomarkers of cellular stress [11,12].

In terms of their origin and biological characteristics, HSPs are extremely conserved ancient proteins present in all organisms from prokaryotes to mammals, including humans [13]. This fact implies the fundamental importance of this biochemical factor group mediated by their chaperone activity: their participation in forming the secondary and tertiary structure of proteins, protein repair processes, as well as in the elimination of pathological and denatured proteins. HSPs are involved in the formation and adaptive stabilization of cellular structures, the restoration of biomolecule conformation, and the regulation of enzyme activity [14,15].

Another important role of HSPs is their interaction with the antioxidant system and the formation of nitric oxide (NO). The connection of HSPs with the metabolism of glucocorticoids, mineralocorticoids, and hormones of the reproductive system was described in some publications [16,17,18]. Despite the fact that some HSPs (those synthesized in adipose tissue) have the properties of adipokines, a few studies analyzed the regulation and possible additional functions of HSPs in adipocytes and adipose tissue. One of the first observations of HSP in adipose tissue focused on the meaning of HSP70 cold induction in brown adipocytes. Cold-induced HSP70 expression is not limited to brown adipose tissue but occurs in blood vessels in a manner dependent on α-1 adrenergic receptor antagonism. Mechanistically, norepinephrine, as a mediator of thermogenesis, is able to induce HSP70 in brown adipocytes potentially through nitric oxide, the so-called NO-dependent pathway [19,20,21].

**Figure 1:**
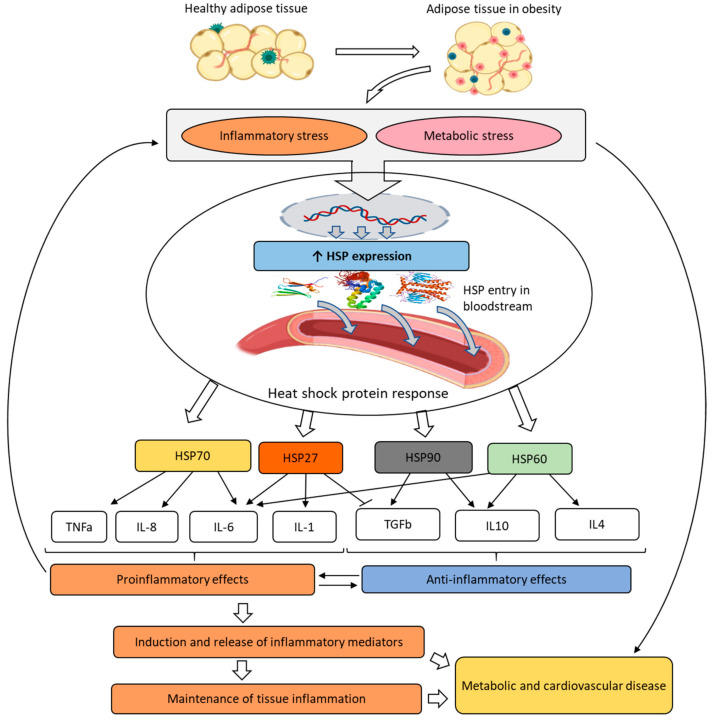
HSP in the pathogenesis of obesity.

A small number of studies linking HSPs to obesity demonstrated variable changes in the levels of these proteins. Some evidence implied elevated levels of HSP60, HSP2, HSP90, and HSP70 in obesity. However, the nature of such changes was multidirectional and depended on the HSP type and the type of analyzed biomaterial [16,22]. It was suggested that disorders in the HSP system may play a significant role in the development of insulin resistance and cardiovascular diseases in obesity [20,23]. The proposed mechanism of the HSP effect on cardiometabolic disorders is presented in Figure 1.

Pathological adipose tissue causes chronic systemic inflammation and metabolic disorders, leading to the development of so-called inflammatory and metabolic stress. Metabolic stress includes conditions that are associated with the occurrence of metabolic diseases associated with obesity, such as insulin resistance, type 2 diabetes, metabolic syndrome and effects on hormone secretion and release, tissue hypoxia, cell swelling, and reactive oxygen species production. This process involves a physiological response to stress conditions, which includes the overexpression and secretion of HSPs. Once in the bloodstream, HSPs participate in the induction and release of inflammatory mediators, thereby contributing to maintaining chronic tissue inflammation and to the subsequent progression of metabolic and cardiovascular diseases.

HSPs are classified according to their molecular weight and functional activity. Based on molecular weight, HSPs are categorized into low-molecular-weight and high-molecular-weight compounds. Accordingly, the nomenclature of HSPs depends on their molecular weight, e.g., HSP27 means a molecular weight of 27 kDa [24,25,26].

The group of small HSPs (sHSPs) includes proteins of relatively small size (from 16 kDa to less than 40 kDa) that are normally present in the cytoplasm and nucleus. This HSP group serves as a cytoskeleton stabilizer and exhibits antioxidant activity in pathological conditions. HSPs prevent the irreversible aggregation of damaged proteins, which is important without the involvement of ATP (ATP-independent pathway), and transport damaged proteins to ATP-dependent chaperones (e.g., high-molecular-weight HSPs, such as HSP70, etc.). HSPs are also capable of inhibiting apoptosis mechanisms and playing a protective role in various pathological conditions, such as atherosclerosis and obesity [27,28,29]. High-molecular-weight HSPs include a group of proteins of certain families: HSP40, HSP60, HSP70, HSP90, HSP100, etc. They have different structures and functions, which will be described in separate subsections of our review.

Based on their functional activity and synthesis, HSPs are conventionally classified into constitutive and inducible. The synthesis of constitutive HSPs proceeds continuously, and their activation does not require specific conditions. The synthesis of inducible HSPs begins after exposure to a damaging factor. Moreover, some HSPs can be both constitutive and inducible, depending on the type of cells and their functional activity [20,25].

Below we analyze individual proteins and their importance in cardiovascular disease and obesity.

### 2.1. HSP27

One of the most important and well-studied small HSPs is HSP27 (also known as heat shock protein β-1, HSPB1). HSP-27 is a constitutive protein expressed in various tissues [28,30].

In muscle cells, including cardiomyocytes, HSP27 is located adjacent to actin microfilaments. It exerts a chaperone-like effect by capping and stabilizing the ends of these microfilaments. Under stress conditions (e.g., increased temperature, surrounding toxic compounds, oxidants, cytokines, growth factors, peptide hormones, etc.), the overexpression of HSP27 and its phosphorylation were observed. In turn, phosphorylated HSP27 changes its oligomeric structure, which leads to the reorganization of microfilaments. Thus, HSP27 performs a protective function, thereby ensuring cell survival under stress conditions [20,31].

The biomarker potential of HSP27 was examined in several studies focusing on its metabolic effects and association with cardiac events. Alterations in HSP27 expression were described in a number of metabolic diseases associated with obesity. For instance, a decrease in the expression of this factor was shown in the adipose tissue of women with gestational diabetes and obesity. It was assumed that an altered HSP27 response may underlie the pathogenesis of the inflammation of visceral adipose tissue and lead to various disorders. 

In a study by Oliva et al., HSP27 levels were reduced in adipose tissue of 12 women with gestational diabetes mellitus treated with insulin vs. the adipose tissue of 12 women with normal glucose tolerance [32]. Other studies established differences in levels of antibodies to HSP27 (anti-HSP27) depending on the metabolic type of obesity. Metabolically healthy obesity was characterized by higher levels of anti-HSP27 vs. groups of people with a normal body mass index (BMI) value, but the antibody titer correlated with BMI [33,34]. 

A more recent anti-HSP27 study demonstrated that serum anti-HSP27 levels were significantly higher in obese patients than in nonobese individuals. In this study, serum anti-HSP27 levels were measured in 993 subjects recruited from the Mashhad Stroke and Heart Atherosclerotic Disorders (MASHAD) cohort study. According to the general linear model, anti-HSP27 concentrations were also directly associated with BMI and lipidemia. Hence, anti-HSP27 levels were associated with cardiovascular disease risk factors and could serve as a possible biomarker used for risk stratification in obese patients [35]. 

A study by Marion et al. showed the prognostic role of HSP27 in a group of 199 patients with atrial fibrillation. In this study, various HSPs (HSP27, HSP70, cvHSP, and HSP60) and clinical characteristics of patients, including BMI and lipidemic status, were analyzed. Only the basal HSP27 content increased in the serum of patients within a year after ablation therapy, while elevated HSP27 levels were associated with an increased incidence of recurrent atrial fibrillation. This study revealed no association between patients’ BMI and HSP27 levels, even though patients with atrial fibrillation had a significantly higher BMI than control group subjects [17].

It is well known that obesity is one of the essential factors in the development of coronary artery disease (CAD). A recent study by Abaspour et al. demonstrated an association between the number of HSP27 mRNA copies in the mononuclear cells of peripheral blood and the severity of CAD, but no significant differences were detected for BMI and hip circumference between the groups of patients and levels of HSP27. The patients (*n* = 103) were distributed among two groups: subjects with stenosis < 50% and subjects with stenosis ≥ 50%. The expression of the analyzed factor was significantly elevated in both groups vs. the control group; however, in the group with stenosis ≥ 50%, it statistically significantly correlated with the severity of CAD. This finding implied that HSP27 may indicate the degree of general oxidative stress in patients and, consequently, be used as a potential prognostic biomarker of CAD [36].

Hence, although HSP27 is involved in the essential cellular processes occurring in conditions of obesity or its cardiometabolic complications, its use as a biomarker has been studied very little and requires more in-depth investigation. On the one hand, HSP27 has protective properties and its hypersecretion may indicate the relevance of the cellular response to stress; on the other hand, the very development of such cellular stress may constitute a risk factor [37,38,39]. 

### 2.2. HSP40

HSP40 (also known as heme oxygenase 1, HO-1) is a member of the DnaJ family of proteins, the distinctive features of which are the presence of a J-domain responsible for the activation of the HSPs of the HSPA family (including HSP70) and the stimulation of the activity of ATPases and other co-chaperones [40]. HSP-40 promotes protein remodeling via the sequential folding and refolding of protein aggregates, and also contributes to the maintenance and transport of collagen. Proteomic studies demonstrated that HSP40 is included in the secretome of human adipocytes, which allows us to classify it as an adipokine [25,40]. The adipokine nature of HSP40 requires an analysis of its potential as a biomarker in terms of the cardiovascular complications of obesity. Unfortunately, current published sources do not provide noteworthy direct evidence of a connection between HSP40 and the cardiovascular complications of obesity. However, we found some evidence linking HSP40 to obesity, insulin resistance, and physical activity, all of which are considered cardiovascular risk factors [41,42].

Overall, the role of HSP40 in obesity remains highly controversial. This factor is induced by various stimuli, including hypoxia, inflammation, and the mechanical damage of tissues. HSP40 levels in serum and in subcutaneous and visceral adipose tissues are higher in obese patients than in normal-weight individuals. Most importantly, HSP40 expression was higher in obese patients with insulin resistance than in obese but insulin-sensitive patients [41].

On the other hand, there is evidence of a decrease in HSP40 activity in obese patients. According to Abubaker et al., HSP40 expression was reduced in obese individuals. This study examined two groups of adult men and women: a lean group (*n* = 54; BMI = 20–24.9 kg/m^2^) and an obese group (*n* = 66; BMI = 30–40 kg/m^2^). The authors discovered that HSP40/DNAJB3 cochaperone mRNA and protein in obesity were inversely associated with body fat percentage, triglycerides, and inflammatory chemokines. However, the expression of this protein in the studied group of patients increased after they exercised [43].

Therefore, the available data do not lead to conclusions about the potential of HSP40 as a biomarker for the cardiovascular complications of obesity, but rather demonstrate its controversial role in the pathogenesis of this condition.

### 2.3. HSP60

HSP60 is a molecular chaperone involved in the post-translational modification of proteins in mitochondria and the degradation of denatured proteins. HSP60 was found predominantly in mitochondria, and less frequently on the cell surface or in the extracellular medium. In blood serum, HSP60 is normally found in a physiological state, and its concentration decreases with age. Depending on its localization, HSP60 performs various physiological functions, from regulating the activity of mitochondrial chaperones to participating in the processes of cell proliferation, apoptosis, migration, and immune response [44].

HSP60 is encoded by the nuclear genome and expressed in the cytosol as a precursor called naïve HSP60. In mitochondria, this molecule is transformed into the mature form of mitochondrial HSP60 (mtHSP60). Importantly, the mitochondrial form of HSP60 is less stable due to higher biochemical activity. At the same time, naïve HSP60 is more stable and can be present in biological fluids (such as blood serum) in the form of oligomeric complexes [45].

HSP60 is one of the most thoroughly studied HSPs in adipose tissue and adipocytes. This factor can be considered an adipokine because it is secreted by adipocytes in measurable amounts. HSP60 expression in stromovascular cells is lower than in mature adipocytes, suggesting that HSP60 is more characteristic of mature adipose tissue cells [46]. There is experimental evidence that HSP60 deficiency leads to mitochondrial dysfunction and, as a consequence, cardiovascular pathology, including dilated cardiomyopathy and heart failure; however, it can stimulate the apoptosis of cardiomyocytes and induce the development of atherosclerosis in the early stages [47,48].

The findings of HSP60 overexpression in adipose tissue are consistent with measurements of circulating concentrations of this protein, which are higher in the serum of obese individuals. It was suggested that HSP60 may be activated in adipocytes by proinflammatory stress signals, such as lipopolysaccharides, IL-1β, and TNFα [49]. In turn, the effect of HSP60 on adipocytes leads to the release of proinflammatory cytokines and adipokines. Thus, HSP60 could be a component of the formation of chronic inflammatory responses associated with obesity, and could therefore be considered as a link between obesity, chronic inflammation, and the development of cardiometabolic disorders [20,50].

Sell et al. demonstrated an association between HSP60 levels and obesity dynamics during bariatric surgery in a group of 53 female patients with obesity. The serum HSP60 levels were significantly increased in the morbidly obese patients (31.6± 26.8 ng/mL) compared with the lean control individuals (12.6 ± 11.0 ng/mL). The HSP60 levels decreased following surgery-related weight loss from 31.6 ± 26.8 ng/mL to 21.1 ± 18.7 ng/mL at 12 months postoperatively. Also, the concentrations of the analyzed protein directly correlated with the levels of triglycerides, apolipoprotein B (ApoB), glycated hemoglobin, and CRP. Hence, HSP60 reflected the disease dynamics and demonstrated an association with lipid metabolism and inflammation [51]. 

Based on a group of 129 hypertensive and 39 normotensive women, a study by Kuka et al. analyzed the association between plasma HSP60 levels, hypertension, oxidative stress, lipid profile, and cardiometabolic risk factors, including abdominal obesity, metabolic syndrome, and diabetes mellitus. The authors established an association between blood pressure and HSP60 in the hypertensive group, and between HSP60 levels and total glutathione in the normotensive group [52].

The study by Damluji et al. revealed that elevated anti-HSP60 concentrations were associated with increased coronary artery calcium (a marker of atherosclerosis in asymptomatic adults), but no differences in the levels of this antibody were influenced by diabetes, hypertension, obesity, or dyslipidemia. These findings suggested the potential use of serum anti-HSP60 as an independent biomarker of the early development of atherosclerosis in asymptomatic obese patients [53].

Khadir et al. conducted a study involving obese adult men (*n* = 120) and women (*n* = 110) divided into two groups, without (*n* = 138) and with diabetes (*n* = 92). The study demonstrated that HSP60 mRNA and HSP60 protein levels were reduced in the subcutaneous adipose tissue of obese diabetic patients, along with an increased expression of inflammatory markers and glycemic levels, vs. obese adults without diabetes. After three months of exercise, HSP60 expression levels increased significantly in the group with diabetes and decreased in the group without it. These results were confirmed by measuring antibodies against HSP60 in subcutaneous adipose tissue, but not in plasma [54].

Interesting results were obtained by Nahas et al. in a study of the relationship between serum HSP60 levels and cardiovascular risk factors in 311 postmenopausal women depending on the presence of metabolic syndrome. The serum HSP60 concentrations were significantly higher in the women with metabolic syndrome and increased with blood pressure, triglyceride levels, and other risk factors. Therefore, the results of this study suggested that high serum HSP60 levels were associated with cardiovascular risk in postmenopausal women with metabolic syndrome [55].

HSP60 is released into the extracellular medium during necrosis and is an important cell death signal. Thus, HSP60 is involved in the pathogenesis of atherosclerosis, rheumatoid arthritis, diabetes mellitus, and neurological diseases [25]. In a more recent study by Yıldırım et al., serum HSP60 levels were analyzed in 40 patients with obesity-related kidney disease vs. 40 obese patients without such complications and 40 nonobese individuals (the control groups). The study showed a significant increase in the mean concentration of HSP60 in the blood serum of patients with kidney diseases associated with obesity (537.6 ± 170.4 ng/mL) vs. control groups with a normal body weight (371.8 ± 76.3 ng/mL) and with obesity (430.8 ± 110.6 ng/mL), while HSP60 was associated not only with the presence of obesity-related kidney disease (ORKD) but also with the total protein in the urine [56].

Hence, HSP60 is involved in various pathological processes in the cardiovascular, immune, excretory, and nervous systems, and is associated with obesity and related diseases. The hypersecretion of HSP60 and antibodies to it is, in most cases, associated with a poor prognosis, but the role of HSP60 in tissues is more controversial. Given that HSP60 concentrations are measurable in serum, this protein may become an accessible target for further research in the field of obesity and its cardiac complications.

### 2.4. HSP70 and HSP72

HSP70 is a member of the HSP family with a molecular weight ranging from 66 to 78 kDa. It is a dimeric protein with eight isoforms capable of forming oligomeric complexes that include various cellular structures [57]. HSP70 is an inducible protein, the expression of which is upregulated under conditions of cellular stress, and is involved in cell repair, survival, and the prevention of protein aggregation. Under cellular stress, HSP70 rapidly accumulates first in the nucleus and then in the cytoplasm of cells. Then, HSP70 normalizes the formation of ribosomes, protects protein synthesis, maintains the integrity of the cellular protein structure, and participates in DNA repair processes [25,58].

There is some evidence that intracellular and extracellular HSP70 have different effects [59]. Intracellular HSP70 plays a cytoprotective role, while extracellular HSP70 exhibits proinflammatory activity when acting as an element of immune signaling pathways. High levels of circulating extracellular HSP70 can induce the release of proinflammatory cytokines, including TGF-β1, through the nuclear factor kappa B system (NF-κB) and activator protein 1 (AP-1) [60].

The protective role of intracellular HSP70 was indirectly confirmed in a number of studies. Low levels of HSP70 in pancreatic cells may be associated with their increased vulnerability to cellular stress, impaired insulin secretion and, ultimately, an increased rate of cell death [61]. There is evidence that a reduction in the level of intracellular HSP70 may serve as a risk factor for the development of diabetes mellitus (type 1 or type 2) [62].

Extracellular HSP70 exhibits different relationships. Alemi et al. demonstrated that the extracellular level of HSP70 in obese patients with type 2 diabetes was significantly higher than in nonobese type 2 diabetic patients. Circulating levels of this protein were also associated with diabetes complications and insulin resistance. Protein levels were observed to decline during exercise [63]. 

The results of the study by Islam et al. were different; HSP70 levels were inversely associated with BMI, body fat percentage, waist circumference, and insulin resistance. This study included 54 people distributed among three groups of equal sizes: an obese group, a normal weight group, and an overweight group. HSP70 levels were significantly lower in the obese group, compared with the overweight and normal weight groups; in turn, the level of the studied protein was significantly lower in the overweight group vs. in the lean patients. The authors hypothesized that high cardiovascular risk conditions, such as obesity and diabetes, could be associated with the impaired expression of HSP70 [34]. 

A study by Lubkovskaya et al. included 57 subjects without diabetes divided into two groups: 30 lean men and 27 overweight men. The study demonstrated that young overweight individuals had reduced serum HSP70 levels, compared with lean study participants [64].

In general, the balance between extracellular and intracellular HSP70 may be associated with physical activity, especially in patients with obesity and its metabolic complications [65]. Exercise is a stimulus for increased HSP72 expression. Archer et al. proposed that exercise-induced extracellular release of HSP72 may contribute to the beneficial metabolic effects via the active restoration of HSP72 content in insulin-resistant tissues with low endogenous levels of this protein [66].

In their study on HSP70 expression in various types of cells and tissues of obese patients, Vulf et al. established its statistically significant increase in the mononuclear cells of peripheral blood, compared with nonobese individuals. Notably, the expression level of HSP70 was suppressed in visceral subcutaneous adipose tissue and liver, where the authors observed a high expression of proinflammatory cytokines [67].

Di Naso et al. examined the association of HSP70 levels in liver and visceral adipose tissue with insulin resistance and non-alcoholic fatty liver disease (NAFLD) in obese patients after bariatric surgery. Reduced levels of HSP70 and its transcription factor (HSF1) correlated with insulin resistance and NAFLD progression. The lowest expression was detected in the group with steatohepatitis and liver fibrosis. These data suggest that the disruption of HSP70 and HSF1 may affect anti-inflammatory mechanisms, along with inducing oxidative stress and insulin resistance [68]. Obesity-related asthma was associated with a high content of HSP72 in urine, highlighting the possible role of HSP72 in the development of this complication [69].

Another study linking HSP70 to cardiovascular disease in obesity examined the 1267HSP70-2 genetic polymorphism and established its association with obesity in 317 subjects: 94 obese individuals who underwent coronary angiography and healthy controls (93 obese and 130 nonobese volunteers). The study showed a significant association of HSP70-2 gene + 1267HSP70-2G >A polymorphism with obesity and demonstrated that subjects with the GG genotype or those who carried the G allele were associated with obesity as well. This finding implied that there are deeper pathophysiological mechanisms in which HSP70 is involved [70].

As we can see, the importance of HSP70 as a biomarker is highly controversial. Intracellular HSP70 exhibited the properties of a favorable factor in most studies. Extracellular HSP70, which can be measured in the circulation, is more suitable for use as a noninvasive biomarker, but there is currently no common understanding of its biochemical dynamics in different patient groups, diagnoses, etc. Hence, before discussing its biomarker potential for cardiometabolic complications of obesity, we need more substantial evidence.

### 2.5. HSP90

HSP90 is a member of the HSPC family. HSP90 forms a complex with several auxiliary proteins known as cochaperones. Such complexes interact with steroid hormone receptors and ensure the effective binding of hormones to the receptors and the subsequent transfer of the hormone–receptor complex into the nucleus. Besides this, HSP90 is involved in the targeted transfer of certain types of protein kinases to their functional sites. HSP90 may be involved in the folding of multidomain proteins, such as actin or tubulin, as well as in the ATP-dependent correction of partially denatured proteins [71].

The HSP90 family of proteins is a very ancient type of HSP found in all species. Under heat shock conditions, HSP90 is involved in the reactivation of thermally denatured proteins. Mammalian HSP90 typically exists in a dimeric form functionally similar to HSP70. Other chaperones are often found in HSP90 complexes in the cytoplasm; they form an integral part of the multicomponent chaperone system. HSP90 is involved in many signaling pathways, including glucocorticoid and progesterone receptors, estrogen receptors, and protein kinase receptors. A detailed expression analysis confirmed that HSP90 plays a significant role in normal myogenesis, thereby protecting embryonic cells from adverse environmental factors [25,26,72].

In a study by Balanescu et al., circulating HSP90 isoforms in the serum of obese children exhibited properties as useful biomarkers of NAFLD. The authors measured total HSP90 content, along with the concentrations of the HSP90α and HSP90β isoforms. Solely the total and HSP90β levels were higher in overweight and obese NAFLD patients, whereas there was no difference in HSP90α concentrations [73]. Another study (Skorzynska-Dziduszko et al., 2016) established that increased concentrations of HSP-90α in patients with arterial hypertension may be a compensatory mechanism for impaired nitric oxide bioavailability. The content of HSP90α directly correlated with systolic and diastolic blood pressure; it was significantly higher in patients with arterial hypertension vs. normotensive patients [74]. At present, there is a lack of significant data on HSP90 in cardiovascular disease and/or obesity, as well as at the early stages of disease development. 

## 3. Conclusions

Currently, it can be assumed that HSPs are involved in the pathogenesis of cardiovascular pathology. However, it remains somewhat unclear whether HSP-related changes reflect a causative factor or a consequence of cardiovascular diseases associated with obesity. HPS27, HSP60, and HSP70 are the most well-studied HSPs in terms of cardiovascular and metabolic pathology, but even these proteins require further research. In conclusion, it should be understood that there is currently insufficient evidence regarding the association between HSP and obesity-related cardiovascular events, and the mechanisms by which HSP influences obesity-related conditions are not entirely clear. Thus, before considering HSPs as a biomarker of cardiovascular risk in obesity, we need to gain more information about their involvement in various cardiovascular diagnoses in obese patients.

## Data Availability

Not applicable.
